# Implantable and transcutaneous continuous glucose monitoring system: a randomized cross over trial comparing accuracy, efficacy and acceptance

**DOI:** 10.1007/s40618-021-01624-2

**Published:** 2021-07-01

**Authors:** F. Boscari, M. Vettoretti, F. Cavallin, A. M. L. Amato, A. Uliana, V. Vallone, A. Avogaro, A. Facchinetti, D. Bruttomesso

**Affiliations:** 1 Department of Medicine, University of Padova, Via Giustiniani 2, 35128 Padova, Italy; 2 Department of Information Engineering (DEI), University of Padova, Padova, Italy; 3 Independent Statistician, Solagna, Italy

**Keywords:** Continuous glucose monitoring, Implantable and transcutaneous sensors, Sensors accuracy, Sensors acceptance, Type 1 diabetes, Glycemic control

## Abstract

**Aim:**

To compare accuracy, efficacy and acceptance of implantable and transcutaneous continuous glucose monitoring (CGM) systems.

**Methods:**

In a randomized crossover trial we compared 12 weeks with Eversense implantable sensor (EVS) and 12 weeks with Dexcom G5 transcutaneous sensor (DG5) in terms of accuracy, evaluated as Mean Absolute Relative Difference (MARD) vs capillary glucose (SMBG), time of CGM use, adverse events, efficacy (as HbA1c, time in range, time above and below range) and psychological outcomes evaluated with Diabetes Treatment Satisfaction Questionnaire (DTSQ), Glucose Monitoring Satisfaction Survey (GMSS), Hypoglycemia Fear Survey (HFS2), Diabetes Distress Scale (DDS).

**Results:**

16 subjects (13 males, 48.8 ± 10.1 years, HbA1c 55.8 ± 7.9 mmol/mol, mean ± SD) completed the study. DG5 was used more than EVS [percentage of use 95.7 ± 3.6% vs 93.5 ± 4.3% (*p* = 0.02)]. MARD was better with EVS (12.2 ± 11.5% vs. 13.1 ± 14.7%, *p*< 0.001). No differences were found in HbA1c. While using EVS time spent in range increased and time spent in hyperglycemia decreased, but these data were not confirmed by analysis of retrofitted data based on SMBG values. EVS reduced perceived distress, without significant changes in other psychological outcomes.

**Conclusions:**

CGM features may affect glycemic control and device acceptance.

**Supplementary Information:**

The online version contains supplementary material available at 10.1007/s40618-021-01624-2.

## Introduction

Continuous glucose monitoring (CGM) improves glycemic control in subjects with type 1 diabetes (T1D), decreasing HbA1c and reducing hypoglycemic events [1–3].

The ability of CGM systems to transmit data to the cloud, so that it can be stored, shared and remotely viewed by the patient and the healthcare provider, makes CGM particularly suitable for virtual consultations. During the COVID-19 pandemic, remote virtual consultation based on shared CGM data has allowed detailed revision of patients’ glycemic control, remote planning of patients’ goals, therefore improving patients’ outcome [4–7].

However, CGM is still used less than expected, especially by adolescents [8]. Furthermore, CGM systems are often used intermittently. Misuse may be connected with problems with reimbursement, physical discomfort, problems with sensor insertion and holding on the skin, concerns about the accuracy of data, interference with sports and daily activities, skin reactions [9].

To foster the use of CGM, devices with increased accuracy and portability have been produced, together with sensors more easy to apply. Furthermore, some CGM have been approved for non-adjunctive use [10, 11].

Most CGM systems sample glucose concentration in the interstitium of the subcutaneous tissue through a transcutaneous needle-sensor connected to a transmitter that sends data to a receiver or a mobile app. They have a lifetime of 7–14 days and the short life span affects adversely patient adherence.

To overcome these limitations and improve patient’s compliance, an implantable sensor has been developed, with a lifetime of up to 180 days [12]. The sensor, inserted subcutaneously in the upper arm, sends data to a removable transmitter worn over the implant and then to the system’s mobile application via Bluetooth. Sensor implantation and removal require a minor surgical procedure, unlike transcutaneous CGM systems, which are self-inserted by the patients.

Clinical studies have shown that both transcutaneous and implantable systems are safe, well tolerated and effective, reducing HbA1c and hypoglycemic events and improving the quality of life [13–16].

However implantable and transcutaneous CGM systems have differences that could influence acceptance by patients and/or influence effectiveness. Thus far, however, no study has compared the two systems. Aim of this work was to identify features of the transcutaneous or implantable CGM systems that affect glycemic control and device acceptance. To that end, we compared in real life the transcutaneous CGM device most used in Italy with the only available implantable system. Accuracy, time of CGM use, efficacy, safety and different psychological aspects were considered.

## Material and methods

### Study design

This was a monocentric randomized crossover study comparing the implantable system Eversense (EVS; Senseonics, Inc., Germantown, MD) with the transcutaneous system Dexcom G5 (DG5; Dexcom, San Diego, CA), a transcutaneous system.

### Participants

Participants were 18 years or older, with a diagnosis of T1D from at least one year (World Health Organization criteria), treated with CSII or MDI, with HbA1c < 10% (86 mmol/mol). Exclusion criteria were episodes of hypoglycemia in the previous 12 months, pregnancy, lactation, medications (apart insulin) affecting glucose metabolism, inability to comply with study procedures, allergy to skin patches or disinfectants. During the study participants omitted drugs interfering with sensor-related measurement of glucose like acetaminophen.

Participants were randomly assigned to 12 weeks with DG5 as first system and then to 12 weeks with EVS (arm AB) or vice versa (arm BA) in a 1:1 ratio. Randomization was performed using a computer-generated random assignment list.

### CGM systems

#### Implantable sensor

EVS is, at present, the only implantable sensor available. It consists of an implantable fluorescence based sensor, a removable external transmitter and a mobile medical application that displays glucose data. The sensor is inserted in the subcutaneous tissue of the upper arm by a certified medical team, through a small incision and using a special dissector. The transmitter, sitting over the sensor and secured to the skin with an adhesive, stores glucose data and transfers them via Bluetooth Low Energy to the Eversense app on the patient’s smartphone that displays glucose values, trends and alerts. There is a 24-h warm up period after sensor implantation, followed by an initialization period of 12 h during which the sensor need to be calibrated several times. Afterwards the system requires twice-daily calibrations. The transmitter is charged daily and this takes approximately 15 min.

The EVS system alerted the subject during rapid glucose changes and when glucose values exceed or were predicted to exceed a selected threshold. In the absence of a smartphone, subjects could be alerted by the transmitter through a vibration. Subjects could insert in the smartphone app glucose value obtained by Self-monitoring of blood glucose (SMBG), calibration, meals, exercise and insulin.

At the time of this study, the system available in Italy had a declared mean absolute relative difference (MARD) vs venous glucose values of 11.1% and was approved for up to 90 days. It was indicated only for adjunctive use, thus requiring confirmatory SMBG. The new version of the sensor has a MARD of 8.8% and is approved for 180 days use [17, 18].

#### Transcutaneous sensor

As a transcutaneous CGM system, we used the DG5, which utilizes a sensor, based on glucose-oxidase, inserted under the skin by patients and is connected to a transmitter that directly sends data to a receiver or a patient’s smartphone, which displays glucose readings and trends. The warm-up period after sensor insertion is of 2 h. The lifetime is 7 days and 2 calibrations per day are needed. DG5 gives alarms in case of glucose levels exceeding preset thresholds and in case of rapid glucose changes. It does not have predictive alerts.

Its accuracy has been evaluated in different studies with MARD of 9% vs venous glucose values and it is approved for non-adjunctive use [19, 20], replacing SMBG when users have to make decisions about insulin dosage or hypo treatment.

For both systems retrospective data could be downloaded to generate several glucometric reports.

### Procedures

During the screening visit, inclusion and exclusion criteria were evaluated, and study procedures were illustrated to eligible subjects. Participants were then randomized to AB or BA arm and subsequent visits were scheduled. Participants in AB arm were assigned to wear EVS for 12 weeks followed by DG5 for 12 weeks. Participants in BA arm were assigned to the reverse sequence.

At the beginning of the study and at the end of each study phase, a blood sample was taken to determine Glycated hemoglobin (HbA1c) at the University Hospital of Padova certified laboratory. After randomization, participants were instructed to interpret alarms and to calibrate the G5 and Eversense twice daily as per the manufacturer’s instructions using Accu-Chek Aviva (Roche Diagnostics, Mannheim, Germany). In addition to calibrations, subjects were asked to check blood glucose (BG) values anytime they thought the sensor readings were not consistent with what they expected. In the event of a premature G5 sensor failure, participants were provided with a spare G5 sensor. If the EVS sensor failed prematurely, participants were asked to contact our center for a quick replacement. In both cases, participants were asked to record unscheduled sensor insertions. Subjects came to the clinical center monthly (28 ± 2 days) to download data from meter and sensor receiver. Sensor application site was inspected for skin reactions or adverse local events.

At the end of each study phase (A or B), the sensor was removed and data were downloaded. Participants had no limitations in physical activity but were asked to maintain similar activity levels during the 2 phases of the study. They were free to modify glucose threshold alarms to optimize glucose control and, while using DG5, they could choose between using a receiver or mobile app, applying DG5 sensor on the abdomen as specified in technical sheets. They were free to perform SMBG as usual, with a study meter. In case of transmitter failure, the transmitter was changed if failure happened during the first 8 weeks of the study, otherwise the study was stopped and data were analyzed. All DG5 sensor failures were registered. If EVS sensor failed, sensor was changed only if a failure happened in the first 4 weeks of use; if sensor failed between 4 and 8 weeks of study the participant was excluded from the study; if sensor failed between 9 and 12 weeks available data were analyzed. Questionnaires were administered at the beginning of the study and at the end of each study phase.

### Outcomes

#### Primary outcomes

The primary outcome was sensor accuracy, expressed as mean absolute relative difference (MARD) versus capillary glucose values obtained by SMBG. Since CGM and reference values could not always be obtained at the same time, CGM values were linearly interpolated to match reference values. When a glucose value was used for calibration, it was matched with the previous sensor reading, to avoid sensor readings being affected by calibration. Accuracy was evaluated both overall glucose values and after dividing them into ranges: hypoglycemic (< 3.9 mmol/l, < 70 mg/dl), hyperglycemic (> 10 mmol/l, > 180 mg/dl) and euglycemic (3.9–10 mmol/l, 70–180 mg/dl). Sensor accuracy was also assessed by calculating the percentage of data points falling into zones A and A + B of the Clarke Error Grid (CEG). Values fulfilling ISO 15,197:2013 criteria were expressed as percentage of sensor data within ± 15% of reference value for glucose concentrations ≥ 5.6 mmol/l (100 mg/dl), and within ± 0.8 mmol/l (15 mg/dl) of reference value for glucose concentrations < 5.6 mmol/l (100 mg/dl) [21].

#### Secondary outcomes

##### Time of CGM use

Time of CGM use was evaluated comparing all data recorded during 12 weeks on EVS or DG5. Furthermore, to evaluate the use of the system by the patient, excluding periods in which sensor-transmitter was not active for technical reasons, we compared data collected with the data the CGM system could theoretically collect (288 values/day). Sensor use was evaluated also in sub periods of 4 weeks each. Sensor or transmitter failures were recorded.

##### Efficacy

Efficacy was evaluated as changes in HbA1c and as percentage time spent in the hypoglycemic (< 3.9 mmol/l, < 70 mg/dl), hyperglycemic (> 10 mmol/l, > 180 mg/dl) and euglycemic (3.9–10 mmol/l, 70–180 mg/dl) ranges. Such metrics were calculated both on the original CGM traces and on the retrofitted CGM traces, i.e. CGM traces post-processed by the retrofit algorithm.

The retrofit algorithm performs a de-noising of CGM data and uses the available SMBG measurements to recalibrate the sensor trace to compensate for possible delays and drifts in time of the CGM signal. The output of the retrofit algorithm is called “retrofitted CGM” profile. Calculation of glycemic control metrics on the retrofitted CGM traces provides a more accurate assessment of glycemic control compared to the metrics calculated on original CGM data, thanks to the cleaning action of the retrofit algorithm [22, 23].

##### Safety

Safety endpoints concerned the use of device and sensor insertion/removal procedure and were checked monthly.

##### Psychological outcomes

Psychological outcomes were evaluated with validated questionnaires administered to participants at the beginning and end of each study phase.

The emotional status was assessed with the *Diabetes Distress Scale (DDS)* that is a 17-item scale assessing worries and concerns specifically related to diabetes. It includes 4 subscales [emotional burden, 5 items (EB), physician-related distress subscale, 4 items (PD), regimen related distress subscale, 5 items (RD), diabetes-related interpersonal distress subscale, 3 items (ID)] [24, 25]. Patients rated the degree to which each item was currently problematic for them on a 6-point Likert scale, from 1 (no problem) to 6 (serious problem). Higher mean score indicates greater stress perceived by patients.

The diabetes treatment satisfaction was evaluated using the *Diabetes Treatment Satisfaction Quality (DTSQ)* [26, 27] made of eight items with a scoring range of 0–6 points; six items are summed to produce a measure of treatment satisfaction (higher score indicating higher satisfaction), while the remaining two items are evaluated individually to explore the perceived frequency of hyperglycemic and hypoglycemic episodes. For these two items, low scores represent lower hypo and hyperglycemia perception.

The satisfaction was evaluated using the *Glucose Monitoring Satisfaction Survey, type 1 version (GMSS)* [28] that evaluated device satisfaction through a 5-point Likert scale with a greater value denoting higher satisfaction or less hassle.

Patients’ fear of hypoglycemia was assessed using the *Hypoglycemia Fear Survey (HFS-II)* [29], that is a 33-item questionnaire on a 5-point Likert scale (0–4). It is composed of two subscales assessing behaviors (HFS-B, 15 items) and worries (HSF-W, 18 items) related to fear of hypoglycemia. Higher total scores indicate greater fear of hypoglycaemia.

All questionnaires were validated in Italian, using a forward-back translation procedure.

At the end of the study, participants were also asked to report their opinion through a questionnaire that compared sensors’ features.

### Sample size

A minimum of 12 participants was required to have an 80% chance of detecting at the 5% level, a standardized effect size of 1.25 in a crossover design. The sample size was finally set at 16 participants (8 in AB arm and 8 in BA arm) to take into account possible dropouts.

### Statistical analysis

Continuous data were expressed as mean and standard deviation (SD) or median and interquartile range (IQR), and categorical data as frequency and percentage. Accuracy, sensor use, quality of life and efficacy were compared between sensors using the paired Student *t*-test and the paired Wilcoxon signed-rank test to compare normally and not normally distributed variables, respectively. Participant’s opinion about sensors was summarized for descriptive purpose. All tests were 2-sided and a p-value less than 0.05 was considered significant. Statistical analysis was performed using the Statistics Toolbox of MATLAB (Release 2017a, The MathWorks, Inc., Natick, Massachusetts, United States) and R 3.5 (R Foundation for Statistical Computing, Vienna, Austria) [30].

## Results

Sixteen subjects were enrolled and randomized to EVS-DG5 (8 subjects) or DG5-EVS (8 subjects). All participants, whose characteristics are described in Table 1, completed the study. Eight patients were using the flash glucose monitoring system before entering the study. During the study mean number of SMBG measurement per day was 4.84 (SD 1.65) in EVS and 4.33 (SD 1.39) in DG5 (*p* = 0.029).

**Table 1:** Participant characteristics

Characteristics	All participants	Arm: EVS-DG5	Arm: DG5-EVS
Participants, *n*	16	8	8
Males, *n* (%)	13 (81%)	6 (75%)	7 (88%)
Age (years), mean (SD)	48.8 (10.1)	51.8 (7.7)	45.9 (11.8)
Diabetes duration (years), mean (SD)	29.8 (10.1)	34.3 (9.6)	25.1 (8.9)
BMI (kg/m^2^), mean (SD)	27.6 (3.8)	27.3 (1.7)	27.8 (5.3)
HbA1c (%), mean (SD)	7.4 (0.8)	7.4 (0.8)	7.4 (0.9)
HbA1c (mmol/mol), mean (SD)	57.3 (9.1)	57.0 (8.4)	57.5 (10.3)
Therapy (CSII), *n* (%)	12 (75%)	8 (100%)	4 (50%)
Subjects using SMBG before study, *n* (%)	8 (50%)	5 (63%)	3 (37%)
Subjects using FGM before study, *n* (%)	8 (50%)	3 (37%)	5 (63%)

Data are expressed as Mean (Standard Deviation) or number of subjects (percentage)

*BMI* body mass index, *CSII* continuous subcutaneous insulin infusion, *SMBG* self-monitoring blood glucose, *FGM* flash glucose monitoring, *EVS* Eversense, *DG5* Dexcom G5

### Accuracy

Overall, EVS performed better than DG5 with a MARD vs. SMBG of 12.27% ± 11.55% (mean ± SD) vs. 13.14% ± 14.76%, p-value < 0.001. When accuracy was evaluated in different glucose ranges, EVS was more accurate than DG5 in the euglycemic range, while there was no difference in the hypo- and hyper-glycemic ranges (Table 2). EVS accuracy did not change during the length of the study (Supplementary material 1).

**Table 2:** Sensor accuracy, all pairs analysis

	DG5 vs SMBG	EVS vs SMBG	*p* value
Data pairs (*n*)			
Overall	5001	5099	
Hypoglycemia (< 70 mg/dl)	177	240	
Euglycemia (70–180 mg/dl)	3227	3447	
Hyperglycemia (> 180 mg/dl)	1597	1412	
MARD, % (SD)			
Overall	13.14 (14.76)	12.27 (11.55)	< 0.001
Hypoglycemia (< 70 mg/dl)	20.99 (46.36)	20.91 (21.06)	0.51
Euglycemia (70–180 mg/dl)	13.24 (12.79)	12.05 (11.38)	< 0.001
Hyperglycemia (> 180 mg/dl)	12.05 (10.33)	11.34 (8.79)	0.39
Data pairs in zone A of CEG %			
Overall	80.38	81.86	
Hypoglycemia (< 70 mg/dl)	80.79	74.17	
Euglycemia (70–180 mg/dl)	78.71	81.38	
Hyperglycemia (> 180 mg/dl)	83.72	84.35	
Data pairs in Zone A + B of CEG %			
Overall	98.66	98.47	
Hypoglycemia (< 70 mg/dl)	80.79	74.17	
Euglycemia (70–180 mg/dl)	99.75	99.80	
Hyperglycemia (> 180 mg/dl)	98.43	99.36	
ISO 15/15%			
Overall	69.53	72.07	
Hypoglycemia (< 70 mg/dl)	78.53	70.83	
Euglycemia (70–180 mg/dl)	68.95	72.99	
Hyperglycemia (> 180 mg/dl)	70.17	69.69	

Accuracy metrics are computed on all CGM-SMBG data pairs available

### Time of sensor use

Five premature EVS transmitter failures occurred: one in a participant during the first 4 weeks of use (with loss of 2 days of data until the transmitter was replaced), two in two different participants during the 4–8 weeks period (with a loss of data of 13 days/patient) and two failures in the same participant (with a loss of data of 9 days).

One premature EVS sensor failure was registered in a participant after 79 days of use.

One premature DG5 transmitter failure occurred in one participant after 75 days.

During the 12-week comparison period, DG5 recorded more data than EVS (Table 3). Mean overall registered data were 23,005.5 (974.9) for DG5 and 21,893.6 (1794.4) for EVS (*p* = 0.01). Percentage of use when the sensor was active (without considering data lost for transmitter failure) was 95.7% (3.6%) for DG5 and 93.5% (4.3%) for EVS (*p* = 0.02).

**Table 3:** Total data collected during DG5 and EVS use and percentage of use when sensor was active

Metric	Sensor use			
	DG5	EVS	Mean difference (95% confidence interval)	*p* value
Total data	23,005.5 (974.9)	21,893.6 (1794.4)	1111.9 (202.5–2021.4)	0.019
% use	95.7 (3.6)	93.5 (4.3)	2.2 (0.3–4.2)	0.025
% use 0–4 weeks	96.1 (3.2)	94.5 (3.3)	1.6 (– 0.05 to 4.2)	0.056
% use 5–8 weeks	95.3 (4.6)	94.2 (4.4)	1.1 (– 0.8 to 3.0)	0.255
% use 9–12 weeks	95.8 (3.5)	91.6 (6.2)	4.2 (1.2–7.1)	0.008

Data are expressed as mean (SD)

The percentage of time spent using DG5 remained constant over time, while a significant reduction of EVS use was observed during the 9–12 weeks period (Table 3).

### Efficacy

At baseline, HbA1c was 7.4 ± 0.8% in the EVS-DG5 arm and 7.4 ± 0,9% in the DG5-EVS arm. At the end of the study, HbA1c was 7.0 ± 0.6% for EVS-DG5 and 7.1% ± 0.8% for DG5-EVS (*p* = 0.53).

During EVS use there was an increase of the time spent in the target range and a decrease of the time spent in hyperglycemia, with lower mean glucose level and standard deviation with respect to DG5 (Table 4). Analysis of retrofitted data based on SMBG values confirmed the results only about mean glucose level, standard deviation and time spent above 250 mg/dl.

**Table 4:** Glycemic control during EVS and DG5 sensor use

Metric	DG5	EVS	*p* value
*Metabolic control (registered data)*			
Time in range %	66.99 (11.8)	71.14 (12.29)	**0.028**
Time in hypoglycemia %	2.20 (1.92)	2.57 (2.31)	0.40
Time in hyperglycemia %	30.81 (12.16)	26.29 (12.24)	**0.025**
Time < 54 mg/dl %	0.46 (0.57)	0.53 (0.67)	0.96
Time > 250 mg/dl %	7.22 (5.40)	4.55 (4.30)	**0.0004**
Mean glycaemia [mg/dl]	159.08 (17.15)	152.25 (15.72)	**0.011**
Standard deviation of glycemia [mg/dl]	53.6 (9.99)	48.63 (9.84)	**0.00005**
*Metabolic control (retrofitted data)*			
Time in range %	64.01 (11.96)	67.57 (12.82)	0.068
Time in hypoglycemia %	1.90 (1.71)	2.22 (2.27)	0.30
Time in hyperglycemia %	34.09 (12.34)	30.21 (13.03)	0.062
Time < 54 mg/dl %	0.35 (0.48)	0.47 (0.63)	0.07
Time > 250 mg/dl %	8.05 (5.99)	6.10 (5.68)	**0.04**
Mean glycaemia [mg/dl]	163.14 (17.34)	157.6 (17.21)	**0.049**
Standard deviation of glycemia [mg/dl]	53.8 (9.69)	50.93 (10.13)	**0.00054**

Differences in time spent in glycemic ranges in registered data (upper) and in retrofitted data based on SMBG (bottom). Data are expressed as mean (SD)

### Safety

No adverse events occurred during the study.

### “Patient-reported outcomes” questionnaires

**Fig. 1:**
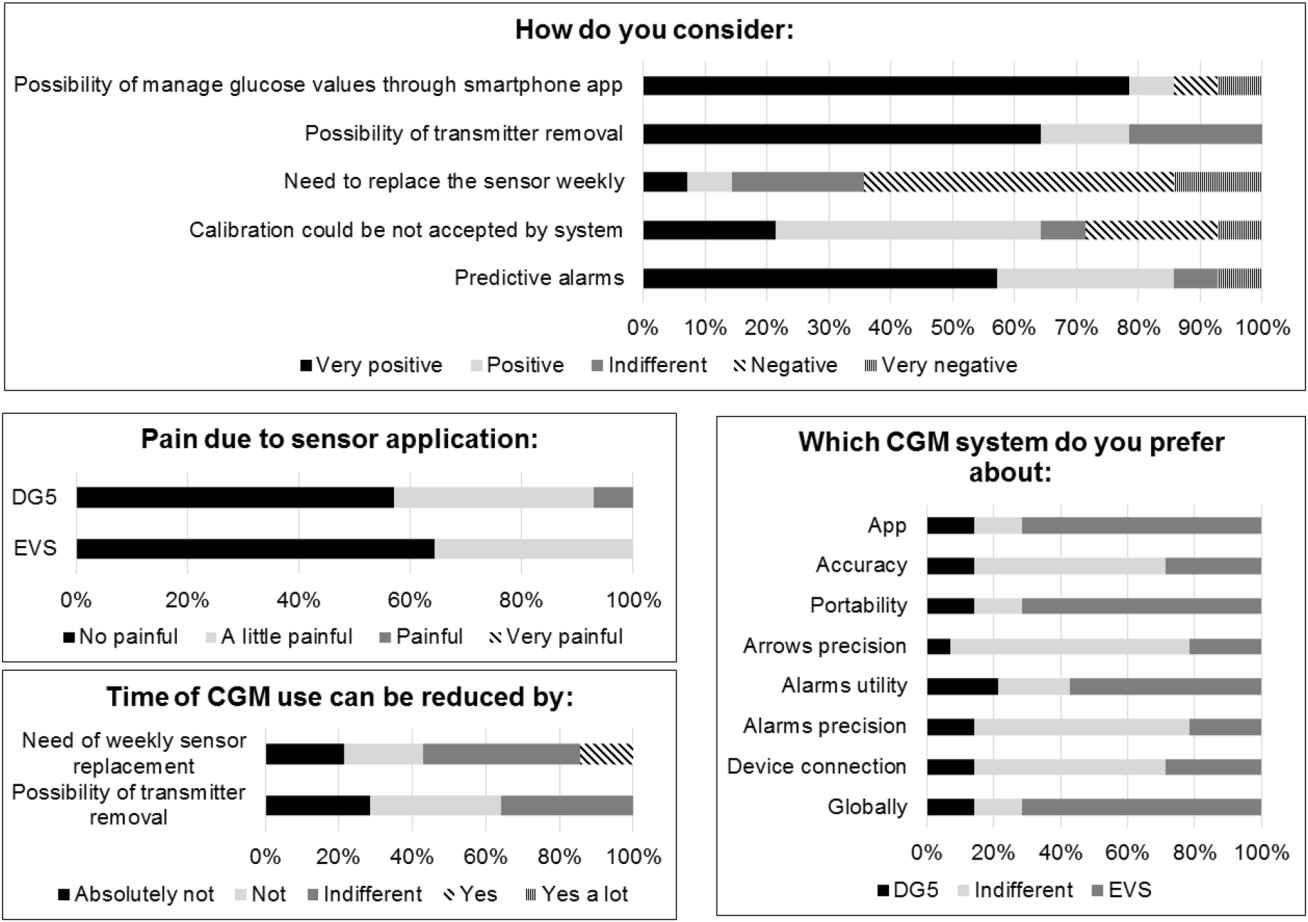
Participant’s opinion about sensors

EVS showed lower scores of DDS total with a mean (SD) score of 2.1 (1.1) vs 2.6 (1.4), p-value 0.009. Emotional burden, regimen-related distress and interpersonal distress subscales demonstrated similar results (Supplementary material 2). No statistically significant differences were found analyzing other questionnaires (Supplementary material 2). Fourteen participants accepted to report their opinion about the sensors (Fig. 1; numerical data in Supplementary Materials 3 and 4). Participants considered sensor application slightly or not at all painful for both sensors. The majority of them considered the possibility to use a Smartphone app to see data preferable with respect to using a receiver, thus preferring EVS app (10 participants). Predictive alarms were considered useful by 12 participants, with a similar degree of satisfaction between sensors about alarms and arrows accuracy. The possibility of transmitter removal or the need for weekly sensor substitution was not considered relevant by patients. Overall, participants preferred EVS features, with special appreciation for app and portability, while no preference of one system over the other was reported regarding accuracy and connection between sensor and transmitter and transmitter/receiver or app.

## Discussion

This study compared in real life the accuracy of a transcutaneous and an implantable CGM system. Both sensors had MARD values higher than previously reported [17, 19]. This is not unexpected since previous studies were done in controlled clinical settings and using as reference venous glucose values rather than values obtained by SMBG. Recently a paper by Jafri et al. [31] confirmed this line of thought, demonstrating that in real life both DG5 and EVS perform worse than in controlled clinical settings. These authors suggested that the discrepancy could be related to the lower accuracy of SMBG meter compared to venous values, errors in calibration or problems (i.e. dirt on fingers) during blood collection by fingerstick.

We found that in real life EVS has greater overall accuracy than DG5, in particular in the euglycemic range, a fact not observed in a previous study lasting 7 days [32], probably due to the short duration of the study. The superior performance of EVS could be related to the fact that it does not allow patients to insert a glucose calibration value if it is too different from values registered by the sensor at the moment of calibration if it is lower than 2.2 mmol/l (40 mg/dl) or higher than 22.2 mmol/l (400 mg/dl). The calibration mode of EVS is seen as negative by only 28% of patients. In addition, in a study comparing EVS and DG5 in real-life conditions, Jafri [31] found that EVS had a better MARD during euglycemia and hyperglycemia but not during hypoglycemia. In Jafri study MARD was higher than in the present study for both sensors, a discrepancy probably explained by differences in study design (sensors were worn simultaneously), glucose range considered (< 70, 70–120, > 120, > 180 mg/dl), meter used and by the fact that EVS values were blinded, possibly influencing calibration accuracy.

We also found that using the implantable sensor, the mass of available data and the percentage of sensor use were smaller compared to DG5. The difference could be related to the need to recharge the transmitter every other day for 15 min, to intercurrent transmitter removal or to problems with the connection between CGM components. Previous studies about the use of EVS in real life revealed that the overall median transmitter wear time was between 84.1% [33] and 83.6% [34], confirming that the possibility to remove the transmitter could shorten the time of sensor use. In our study, the overall transmitter wear time was 93.5 ± 4.3% (mean ± SD), with a trend to a decrease during the last 4 weeks of use. The reason for this decrease remains unclear. It might be due to overconfidence leading to a more frequent removal. This fact should be considered when a CGM is prescribed since subjects characteristics (motivation, working or physical activity…) could limit the correct use of the sensor. On the other hand, the possibility of removing the sensor could be attractive for people who otherwise would not use CGM.

In our experience transmitter failures were more frequent with EVS, but this did not translate into decreased confidence by patients, possibly for the quick substitution of malfunctioning EVS devices assured during the study.

In this study, the use of EVS was associated with better metabolic control, more time in target, lower mean glucose, smaller standard deviation and a shorter time in hyperglycemia. It is now established that the time spent in the target range is the best index of metabolic control in individuals with T1D [35]. However, when CGM data were retrofitted on the basis of SMBG, the increase of the time in range and the decrease of the time in hyperglycemia observed with EVS were not statistically significant. On the other hand, retrofitted data confirmed a decrease of mean glucose and of the time spent above 250 mg/dl with EVS, possibly due to the presence of predictive alarms leading to early correction of impending hyperglycemia. No superiority of EVS could be demonstrated regarding the hypoglycemic risk, possibly for the low hypoglycemic risk in our population or for differences in the threshold set by subjects.

We did not register adverse events linked to sensor use. In particular, there were no infections related to implant/removal of EVS, in agreement with evidence that infections linked to the use of this device are very rare and 3–5 times less frequent than using insulin infusion pumps [36].

Regarding psychological outcomes, EVS was preferred by participants, in particular for the lack of weekly sensor replacement, the smartphone app and the alarms, especially the predictive ones. No differences were found between the two systems in fear of hypoglycemia and satisfaction for therapy, in agreement with other studies showing that both systems improve diabetes treatment satisfaction and reduce the fear of hypoglycemia.

During EVS’s use 3 domains of the DDS (emotional burden, regime-related distress and interpersonal distress) improved. This effect, described by others [37], could be related to particular features of the system such as alarms, app and the possibility of temporarily removing the transmitter.

Our study has limitations. First, we evaluated the first generation of EVS system, which, with respect to present models, is less accurate and has a shorter lifetime. Second, several features of DG5 have been improved, with the arrival on the market of DG6 that does not need calibrations and has a longer lifetime. Third, the study was powered to compare the accuracy between the devices (as primary outcome), while the comparison of secondary outcomes may be hampered by the limited sample size.

## Conclusion

In conclusion, our study reveals how different CGM systems affect, under real-life conditions, glycemic control, sensor use and acceptance by patients. In particular, with respect to DG5, EVS appeared to be superior for accuracy, efficacy and acceptability by patients but had a greater number of transmitter failures and a shorter time of use. Larger studies are necessary to confirm the influence of each CGM system over glycemic control. This study indicates that CGM characteristics need to be taken into account when a device is prescribed.

## Supplementary Information

Below is the link to the electronic supplementary material.

Supplementary file1 (DOCX 16 KB)
