# Supplementary material for: Implantable and transcutaneous continuous glucose monitoring system: a randomized cross over trial comparing accuracy, efficacy and acceptance
Source: J Endocrinol Invest. 2021 Jul 1;45(1):115–24. doi: 10.1007/s40618-021-01624-2 (PMC8246426; doi:10.1007/s40618-021-01624-2)
Supplement: Supplementary file 1 — Supplementary file1 (DOCX 16 KB) [file 40618_2021_1624_MOESM1_ESM.docx]

**SUPPLEMENTARY MATERIAL**

Supplementary material 1. EVS accuracy during different weeks of use.

|  | **Eversense per weeks** | | |
| --- | --- | --- | --- |
|  | **1-4** | **5-8** | **9-12** |
| **Mean ARD (MARD)** | 13,25 | 11,81 | 11,66 |
| **SD** | 12,81 | 10,59 | 11,01 |
| **Median ARD** | 9,65 | 9,04 | 8,67 |
| **25th percentile** | 4,42 | 4,29 | 4,17 |
| **75th percentile** | 18,66 | 16,34 | 15,99 |

Supplementary material 2. Psychological outcomes.

| Questionnaire | Total and subscales scores | DG5: mean (standard deviation) | EVS: mean (standard deviation) | Mean difference (95% confidence interval) | p-value |
| --- | --- | --- | --- | --- | --- |
| Diabetes Distress Scale (DDS) | Total | 2.6 (1.4) | 2.1 (1.1) | 0.5 (0.2 to 0.8) | 0.009 |
|  | Emotional burden | 2.7 (1.4) | 2.1 (1.0) | 0.5 (-0.2 to 0.9) | 0.01 |
|  | Physician-related distress | 2.7 (1.8) | 2.3 (1.6) | 0.4 (-0.1 to 0.9) | 0.12 |
|  | Regimen-related distress | 2.7 (1.3) | 2.1 (1.1) | 0.6 (0.2 to 1.0) | 0.007 |
|  | Interpersonal distress | 2.6 (1.2) | 2.0 (1.1) | 0.6 (0.3 to 0.9) | 0.003 |
| Diabetes Treatment Satisfaction questionnaire (DTSQ) | Total | 31.3 (3.9) | 31.2 (4.3) | 0.1 (-2.3 to 2.4) | 0.96 |
| Glucose monitoring satisfaction survey (GMSS) | Total | 4.0 (0.5) | 4.2 (0.5) | -0.2 (-0.5 to 0.1) | 0.18 |
|  | Openness | 4.0 (0.7) | 4.0 (0.7) | 0.0 (-0.6 to 0.5) | 0.79 |
|  | Emotional Burden | 1.6 (0.6) | 1.6 (0.6) | 0.0 (-0.4 to 0.4) | 0.86 |
|  | Behavioral Burden | 1.9 (0.7) | 1.7 (0.7) | 0.2 (-0.2 to 0.5) | 0.27 |
|  | Trust | 3.7 (1.0) | 4.1 (0.8) | -0.4 (-0.9 to 0.1) | 0.12 |
| Hypoglycemia Fear Survey (HFS) | Total | 24.5 (12.8) | 22.4 (8.9) | 2.1 (-4.8 to 9.0) | 0.36 |
|  | Worry | 10.8 (5.8) | 9.3 (6.8) | 1.5 (-2.4 to 5.5) | 0.18 |
|  | Behavior | 13.8 (8.4) | 13.0 (4.1) | 0.8 (-2.7 to 4.2) | 0.65 |

Supplementary material 3. Participant’s opinion about sensors (14 participants)

| Questions | Answer |
| --- | --- |
| How do you consider EVS sensor application? | Very painful: 0  Painful: 0  A little painful: 5  No painful: 9 |
| How do you consider D5G sensor application? | Very painful: 0  Painful: 1  A little painful: 5  No painful: 8 |
| How do you consider the possibility of manage glucose values through smartphone app? | Very positive: 11  Positive: 1  Indifferent: 0  Negative: 1  Very Negative: 1 |
| How do you consider the possibility of transmitter removal? | Very positive: 9  Positive: 2  Indifferent: 3  Negative: 0  Very Negative: 0 |
| How do you consider the need to replace the sensor weekly? | Very positive: 1  Positive: 1  Indifferent: 3  Negative: 7  Very Negative: 2 |
| How do you consider the fact that the calibration could be not accepted by system? | Very positive: 3  Positive: 6  Indifferent: 1  Negative: 3  Very Negative: 1 |
| How do you consider predictive alarms? | Very positive: 8  Positive: 4  Indifferent: 1  Negative: 0  Very Negative: 1 |
| In your opinion. could the possibility of transmitter removal reduce time of CGM use? | Yes, a lot: 0  Yes: 0  Indifferent: 5  Not a lot: 5  Absolutely not: 4 |
| In your opinion, could the need of weekly sensor replacement reduce time of CGM use? | Yes, a lot: 0  Yes: 2  Indifferent: 6  Not a lot: 3  Absolutely not: 3 |

Supplementary material 4. Participant’s preference about sensors (14 participants)

| Which CGM system do you prefer regarding following features? | DG5 | EVS | Indifferent |
| --- | --- | --- | --- |
| App | 2 | 10 | 2 |
| Accuracy | 2 | 4 | 8 |
| Portability | 2 | 10 | 2 |
| Arrows precision | 1 | 3 | 10 |
| Alarms utility | 3 | 8 | 3 |
| Alarms precision | 2 | 3 | 9 |
| Devices connection | 2 | 4 | 8 |
| Globally | 2 | 10 | 2 |
